# Implicit learning deficit in children with Duchenne muscular dystrophy: Evidence for a cerebellar cognitive impairment?

**DOI:** 10.1371/journal.pone.0191164

**Published:** 2018-01-16

**Authors:** Stefano Vicari, Giorgia Piccini, Eugenio Mercuri, Roberta Battini, Daniela Chieffo, Sara Bulgheroni, Chiara Pecini, Simona Lucibello, Sara Lenzi, Federica Moriconi, Marika Pane, Adele D’Amico, Guja Astrea, Giovanni Baranello, Daria Riva, Giovanni Cioni, Paolo Alfieri

**Affiliations:** 1 Department of Neuroscience, Child Neuropsychiatric Unit, Bambino Gesù Children's Hospital, IRCCS, Rome, Italy; 2 Pediatric Neurology Unit, Catholic University and Nemo Center, Rome, Italy; 3 Department of Developmental Neuroscience, IRCCS Stella Maris, Calambrone (Pisa), Italy; 4 Department of Clinical and Experimental Medicine, University of Pisa, Pisa, Italy; 5 Developmental Neurology Division, IRCCS Fondazione Istituto Neurologico C. Besta, Milan, Italy; 6 Department of Neurosciences, Neuromuscular and Neurodegenerative Diseases Unit, Bambino Gesù Children's Hospital, IRCCS, Rome, Italy; Rosalind Franklin University of Medicine and Science Chicago Medical School, UNITED STATES

## Abstract

This study aimed at comparing implicit sequence learning in individuals affected by Duchenne Muscular Dystrophy without intellectual disability and age-matched typically developing children. A modified version of the Serial Reaction Time task was administered to 32 Duchenne children and 37 controls of comparable chronological age. The Duchenne group showed a reduced rate of implicit learning even if in the absence of global intellectual disability. This finding provides further evidence of the involvement of specific aspects of cognitive function in Duchenne muscular dystrophy and on its possible neurobiological substrate.

## Introduction

Duchenne muscular dystrophy (DMD) is a genetic disorder determined by a single gene mutation on the X chromosome. The effect of this gene mutation is the lack of dystrophin production [1]. Dystrophin is a protein normally expressed in muscles, but different isoforms of this protein, such as Dp140 and Dp71, have also been found in the central nervous system, including the postsynaptic pyramidal cells of cerebral cortex, hippocampus and Purkinje cells in the cerebellum, as suggested by studies conducted in dystrophin deficient mdx-mice [2–5].

Several recent studies have reported that severe learning difficulties are more often associated with mutations in the 3′ end of the gene, involving Dp140 and Dp71 isoforms [6–11], confirming the cognitive deficits reported since Duchenne’s first description (1868) [12–13].

A few papers have suggested a specific link between intellectual disorders and a possible cerebellar dysfunction in DMD with increasing evidence for the involvement of the lateral cerebellum and its connections with cerebral cortex and basal ganglia (cerebro-cerebellar networks) [14].

This is not surprising as in the last thirty years, a number of studies have extended the role of the cerebellum to cognitive functions, such as language, abstract reasoning, emotions and the ability to process logically [15–17].

More specifically, it appears that there is a specific impairment in implicit and procedural learning, as observed in adults with cerebellar lesions, affecting the lateral regions of the cerebellum. The role of the cerebellum in the deficits in implicit learning and procedural learning has also been observed in children with acquired neurological disease [18–20] and developmental dyslexia or intellectual disabilities [21–23]. The cerebellum appears to have an important role in detecting and recognising event sequences and in acquiring and automatizing new cognitive procedures [24–25].

The aim of this study was to examine lateral cerebellar function in children and adolescents with DMD using the modified version of the Serial Reaction Time Task (SRTT), originally developed by Nissen and Bullemer (1987) [26] and already used in the dyslexic population [22]. More specifically, we wished to establish whether the SRTT can detect signs of implicit learning difficulties in a group of children with DMD without intellectual disability, and whether these were related to the mutation site.

## Materials and methods

This study is part of a multicentric project aimed to describe cognitive and executive functions in a large sample of Italian children with DMD without intellectual disability. The study group had previously been evaluated following a Neurocognitive Protocol, which includes a number of tests assessing different aspects, but focusing in particular on executive functions and working memory [27]. Four Italian specialized centres for the diagnosis, management and treatment of neuromuscular disorders, and of DMD in particular, were involved in the study: Bambino Gesù Children’s Hospital in Rome, the Pediatric Neurology Unit and NEMO Centre of Policlinico A. Gemelli in Rome, Stella Maris Foundation in Pisa, and Carlo Besta Neurological Institute in Milan. All the study participants periodically receive clinical assessment in the respective reference centres.

The study was approved by the Ethic Committee of the coordinator center (Bambino Gesù Children’s Hospital), and written informed consent was obtained from study participants’ legal representatives.

### Subjects

Thirty-one boys with genetically defined DMD (mean age 8.2 ± 1.5, age range 6.0–11.6 years, mean nonverbal intelligence quotient–IQ- 103) and 37 age-matched male healthy controls (TD, typical development) (mean age 7.9 ± 1.3, age range 6.1–11.6 years, mean nonverbal IQ 103) were included in the study. These were recruited contacting pupils of a primary school and their parents.

Inclusion criteria were the following: i) DMD boys with a proven mutation in the dystrophin gene; ii); primary school age (6–12 years); iii) no cognitive impairment (IQ <70) nor any associated neuropsychiatric disorders, namely drug-resistant epilepsy, autism spectrum disorder, attention deficit and hyperactivity disorder (ADHD), nor any additional neurosensory deficits (hearing/vision problems); iv) no steroid treatment and/or other experimental drugs starting in the previous six months.

The DMD boys were subdivided according to mutation site: proximal gene mutation (exon 1–44), and distal gene mutation (exon 45 onwards), in order to examine whether the involvement of isoforms Dp 140pc and Dp 71 can influence the functions of the lateral cerebellum.

In order to be sure that all patients could perform the task adequately, only patients with preserved upper limb (minimum score was 3 at manual muscle test, Medical Research Council—MRC—scale, in elbow and wrist flexors and extensors, and thumb abductors of one arm) were included.

### Experimental procedure

Implicit learning was evaluated by means of the SRTT [21, 28]. In this task, the subject seats in front of the screen of a portable computer, where a series of single colored circles (green, blue and red) are displayed centrally. Circles have a diameter of 2.8 cm. The time interval between two successive appearances of the circle, as well as the duration of each single colored stimulus on the screen, vary randomly from 0.5 to 2 s. This experimental procedure, induces the learning of a rhythm, rather than a sequence by avoiding a constant sequence of appearance of the targets (green circles), with fixed time intervals. Five blocks of 75 stimulus-response pairs are presented. Color presentation is random only in block V, (R5), whereas in blocks I–IV (O1-O4) a seven-item sequence (BLUE, GREEN, RED, BLUE, GREEN, BLUE, RED) is repeated 15 times in each block.

The subject was asked to carefully look at the stimuli presented on the computer screen and press the space bar as quickly as possible every time the green circle appeared on the screen; each participant was tested individually. The software automatically recorded the subject’s reaction time (RT), i.e. the time between the stimulus appearance on the screen and the subject’s response. Subjects were not aware of the repeated pattern in the first blocks. If the subject learned the order in which the colors alternated on the screen, then his reaction time in the repeated blocks would gradually decrease from the first to the fourth and, more importantly, it would drastically worsen on the last random block. The comparison between RTs on the last ordered block (O4) and RTs on the random block (R5) is usually taken as a measure of implicit learning. In order to verify whether subjects had gained declarative knowledge of the blocks presented, at the end of the five blocks, each subject was asked whether the color presentation was patterned or not, and was then requested to verbally describe the block sequence. Since the aim of our study was to evaluate only implicit learning memory, the results obtained by 1 DMD boy and 2 TD controls were excluded from the final statistical analysis because a declarative strategy during SRTT was used. Thus, all analyses were conducted on 31 DMD participants and 35 TD participants.

## Statistical analyses

In this study the logistic regression analysis was used to verify the probability that participant may belong to a specific group (in the first analysis DMD *v*s TD, and in the second analysis DMD1 *v*s DMD2, respectively) based on the RT’s difference (R5-O4) between the ordered last block (O4) and the random last block (R5), measure of implicit learning effect. The dependent variable was the Group (dichotomous), while the independent variable was the measure of implicit learning (continuous). In addition, the medians of the RTs of the two groups (DMD vs. TDs) on the SRTT blocks were analyzed by means of a two-way mixed ANOVA with Group as a between factor, and Blocks (O1, O2, O3, O4 and R5) as a within factor.

The medians of the RTs on the SRTT blocks of the following three groups were compared: 1) DMD with a proximal gene mutation (DMD1, N = 13); 2) DMD with a distal gene mutation (DMD2, N = 18); 3) TD participants. Namely, a two-way mixed ANOVA was performed, with Group as a between factor, and Block of task (O1, O2, O3, O4 and R5) as a within factor.

Level of significance was set at p < 0.05. Tukey's post hoc test was used when required.

## Results

**Fig 1:**
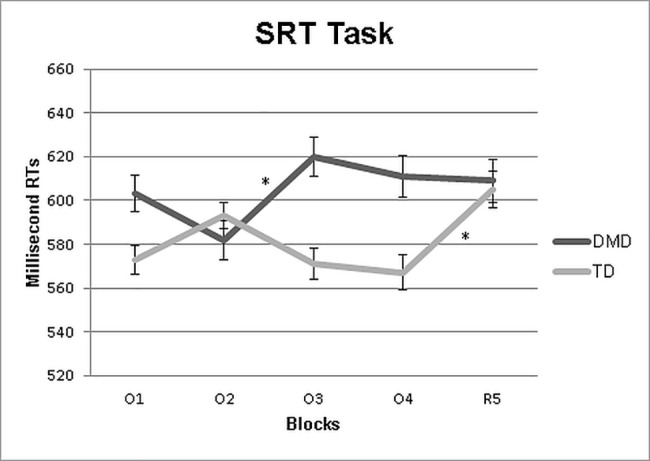
Average reaction times of correct responses of the two groups in all the five blocks of the SRTT. Vertical bars show standard deviation. *p<0.05 from the blocks.

The logistic regression analysis showed that the implicit learning effect significantly predicted (p = 0.03) that a participant belonged to the DMD or TD group (OR = 1.017; 95% CI: 1.006, 1.029). The results of the analysis of RTs by DMD and TD participants on the SRTT blocks did not show a main effect for Group, F(1,63) = 2.9, p = 0.09, as the RTs of DMD participants (M = 608 msec.) did not differ from those of TD participants (M = 578 msec.). The Block effect was instead significant, F(4,252) = 2.7, p = 0.03, indicating longer RTs in R5 than in O4. A crucial result for the aim of this study, is that the Group x Block interaction effect was significant, F(4,252) = 5.57, p = 0.001, thus demonstrating a different pattern of RT changes in the two groups across blocks (Fig 1).

Post-hoc analysis indicated longer RTs in block O3 than in block O2 (p = 0.01) and no difference between O4 and R5 (p = 0.7) in children with DMD, showing the difficulty to learn an ordered sequence. On the contrary, TD controls showed longer RTs in block R5 compared to O1 (p = 0.04), O3 (p = 0.02) and O4 (p<0.001), revealing an appropriate sequence learning effect.

Comparing groups within specific block of the SRTT, the group of DMD and TD did not differ in all blocks (p always>0.1).

Considering the performance of the two DMD subgroups (DMD1 and DMD 2), the logistic regression analysis showed that the implicit learning effect did not significantly predict (p = 0.109) a participant belonged to a specific subgroup (OR = 1.012; 95% CI: 0.997, 1.027).

To confirm this, the analysis focused on the performances of the two DMD subgroups (DMD1 and DMD2) and the TD group failed to show a main effect of Group, F(2,63) = 1.19, p = 0.31: RTs of the two DMD subgroups (DMD 1 = 613 msec.; DMD 2 = 591 msec.) did not differ from each other nor from the TD control group (TD = 578 msec.).

As concerns blocks, a significant Block effect was found F(5,315) = 2.76, p = 0.01, with longer RTs in R5 than O4 (p = 0.01).

**Fig 2:**
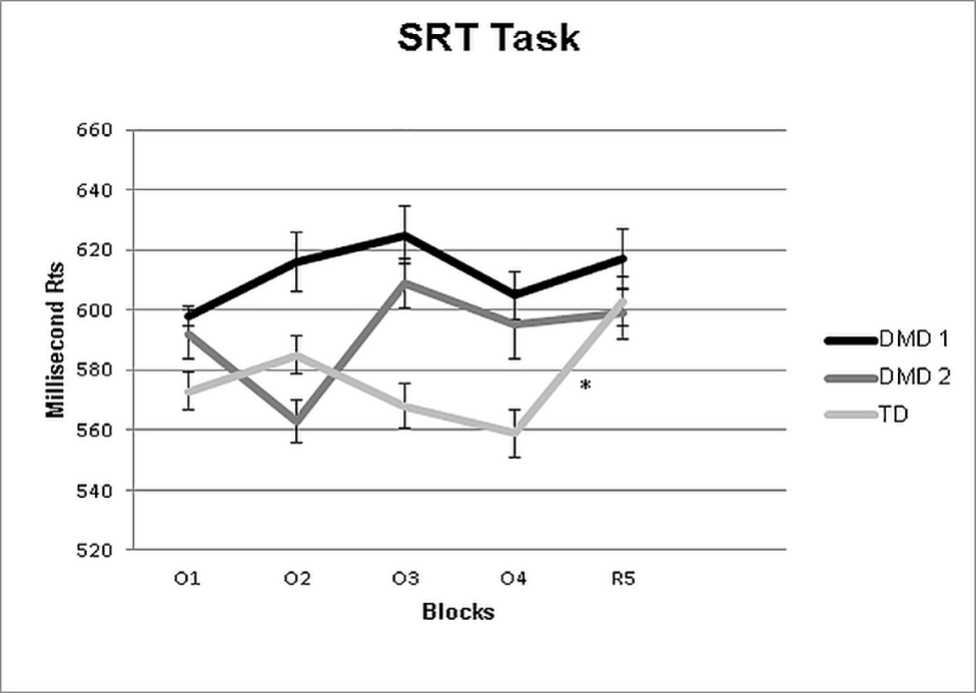
Average reaction times of correct responses of the three groups in all the five blocks of the SRTT. Vertical bars show standard deviation. *p<0.05 from the blocks.

Group x Block interaction was also significant F(10,315) = 2.19, p = 0.01, showing a different pattern of RT changes in the three groups across blocks (Fig 2).

Post-hoc analysis indicated that in the two DMD subgroups, RTs in block O4 did not differ from RTs in block R5 (p always >0.05). On the contrary, in the control group, RT in R5 was longer (p = 0.01) than RT in O4 (O4 = 559 msec. vs R5 = 603 msec.).

## Discussion

In this study, the SRTT was administered to a group of DMD children without intellectual disability and to TD controls in order to investigate their implicit learning and, consequently, their cerebro-cerebellar network function. The exclusion of patients with intellectual disability has enabled us to identify a specific cognitive function, the implicit learning, without the background noise of a more global deficit. Despite having no global intellectual disability, DMD boys revealed poor implicit learning of the temporal sequence of events both in the ordered and in the random blocks of the SRTT while this was not observed in the age matched controls. While in TD controls the reduction of RT across the successive blocks of ordered sequences was largely due to the learning of the temporal sequence of events, in the DMD group, the speed of manual responses was limited, with no or little effects of learning sequence. The comparison between RTs on the last ordered block (O4) and RT on the random block (R5), usually taken as a measure of implicit learning effect, was also different between TD and DMD patients. While, as expected, there was an important change of the reaction time in TD children going from block O4 (ordered) to block R5 (random), this was less obvious in DMD children. The reduced implicit learning observed in DMD children did not seem to depend on their difficulty in manual muscle strength. The results of the logistic regression analysis confirmed that implicit learning effect (R5-O4) characterized the TD group. The similarity of the RTs between TD and DMD, suggested an intact basic motor response, reinforcing the hypothesis of an implicit learning deficit in the group of children with DMD. There were no differences in the overall RTs of the DMD group compared to the TD group, this probably due to the fact that DMD patients with very weak upper limbs (MRC<3) were not included in this study.

It is of interest that between the first (O1) and the second (O2) block, and even more between the second and third blocks (O2 and O3) there were discrepant results between TD and DMD groups. While in TD there was an initial increase of RTs revealing an initial difficulty in the learning ordered sequence (Fig 1), followed by a decrease as the task had been at least partially learned, showing the “U shaped” learning curve usually observed in this type of task [22] (Fig 1); In DMD, in contrast, this was not present. The reduction appeared only after the second block with significantly higher RTs in the third block (O3) compared to the second one (O2). The difficulties in implicit learning was also confirmed by the fact that DMD boys performed similarly in both randomized and ordered blocks, failing to exhibit a learning curve. The performance of three subjects (one DMD subject and two TD controls) were excluded from the statistical analyses because they used declarative strategies. This behaviour could be explained by the length of the ordered sequence of blocks (blocks I-IV; seven sequence elements) that makes it more easily identifiable, especially for older participants. The three subjects excluded from the sample had indeed an average age of 10 years and 5 months.

SRTT is capable to analyze the implicit sequence learning and to demonstrate the role of the cerebellum and its circuits as a key structure for this function. Recent study strongly supports these data, reporting severe impairment in the implicit motor learning task in patients with cortical degeneration of the cerebellum and with focal posterior cerebellar lesions [25, 29–34]. Similar findings on the role of cerebellar circuits in implicit learning were reported also in children and adolescents with Williams syndrome, but not with Down syndrome [35]. In addition, functional MRI studies documented the role of the cerebellum in performing the SRTT in normal readers and dyslexic adults [23]. Finally, as stated in a recent study by Schara and colleagues, subjects with DMD would have a specific cerebellar deficit rather than a global one, with the integrity of the intermediate cerebellum versus a prevalent involvement of the lateral cerebellum [36].

These authors specifically tested cerebellar-dependent delay eyeblink conditioning (a form of implicit associative learning obtained when a neutral conditioned stimulus is repeatedly paired with an unconditioned stimulus to develop a learned conditional response) in eight children with DMD to assess their cerebellar functions. Their results showed a comparable delay eyeblink conditioning in both DMD and controls, thus suggesting a preserved associative learning effect. Because eyeblink conditioning is related to the integrity of the intermediate cerebellum, the authors concluded that this older part of the cerebellum may be relatively preserved in DMD, and suggested, also in line with animal model studies, that the newer, lateral cerebellum is primarily affected in DMD.

The role of cerebellar circuit dysfunctions in implicit learning is however still under debate. Cyrulnik (2008) hypothesized that cognitive deficits observed in DMD could be associated with the absence of dystrophy during the development of the central nervous system, affecting in particular cerebro-cerebellar pathways [14]. Functional imaging studies have shown that a more complex interaction between premotor cortical areas, basal ganglia, and cerebellum underlies to implicit motor sequence learning [37–41]. Moreover, other authors demonstrated that lesions restricted to only one element of this network were not able to impact implicit learning; but, if the basal ganglia damage was associated to frontal cortical areas, as frequently occurred in Parkinson’s disease and Huntington’s disease, the deficit was obvious [42]. This issue is very interesting in DMD, in relation to the absence of dystrophin in the cerebral cortex, especially in the deep layers of the frontal cortex besides other areas such as hippocampus, and cerebellum [3–4].

Investigations of cognitive, language and behavioral problems in DMD individuals and of genotype/phenotype correlations have been already reported [8, 43]. We speculate that deficits in implicit learning abilities may have some clinical manifestations in DMD patients. A recent research conducted by IRCCS Stella Maris group showed the presence of literacy problems in boys with DMD. These individuals showed a similar profile of that present in children with developmental dyslexia (DD), characterized by reading and writing difficulty as well as a delay in automatic naming skills. Also, both children with DD and DMD showed difficulty in representation, accumulation, and recovery of speech sounds [44–45]. Given the overlapping of literacy and phonological deficit in these two groups of children, but also of their reduced procedural abilities [22], it could be suggested a general lack of implicit learning underlying this peculiar neuropsychological profile.

As already discussed, implicit learning plays a key role not only in the acquisition of motor skills but also in the development of cognitive and language abilities such as phonological processing and literacy skills, allowing the automation of reading and writing processes. This may explain the difficulties exhibited by individuals with DMD such as phonological failure [45], general learning difficulties [44–45] and attentional deficits [46–48].

In our study we are also interested to investigate any genotype-phenotype correlation.

Several previous studies documented cognitive and behavioural problems more frequently in association with distal deletion (exon 45 onwards) of the DMD gene [46, 49–50]. In our sample we explored this possible association relatively to implicit learning and in order to minimize the effect of the intellectual disability, we subdivided our sample in two subgroups, both without intellectual disability: first group with a proximal gene mutation (DMD1, N = 13) and the other group with a distal gene mutation (DMD2, N = 18). Our results failed to show any differences between the two subgroups, confirming the hypothesis of an implicit learning deficit in all our children with DMD and emphasizing the possible role of dystrophin in the brain. The results of the logistic regression analysis confirmed that the implicit learning effect (R5-O4) did not significantly predict that a participant belonged to the DMD1 or DMD2 subgroup. However, we cannot exclude the possibility that these results could be attributed to the choice of inclusion criteria of our study, which excluded all participants with intellectual disability, often associated with distal mutations. For these reasons, further work in the future should compare the performance of implicit learning between participants with proximal and distal mutation presenting or not intellectual disability, but matched for their mental age.

In conclusion, our study documented a deficit in implicit learning in a sample of boys with DMD without intellectual disability. On the basis of our knowledge, this deficit may be interpreted as the expression of a dysfunction of the cerebellum and, more specifically, of the lateral regions of the cerebellum and its networks connections.

## Supporting information

S1 Dataset Minimal Manuscript dataset providing group, mutation site, type of mutation, age, non verbal IQ and reaction time (millisecond) on SRTT blocks. (DOCX)
