# Supplementary material for: Implicit learning deficit in children with Duchenne muscular dystrophy: Evidence for a cerebellar cognitive impairment?
Source: PLoS One. 2018 Jan 16;13(1):e0191164. doi: 10.1371/journal.pone.0191164 (PMC5770044; doi:10.1371/journal.pone.0191164)
Supplement: S1 Dataset — (DOCX) [file pone.0191164.s001.docx]

| **Group** | **Mutation site** | **Type of mutation** | **Non verbal IQ** | **P1Mdn** | **P2Mdn** | **P3Mdn** | **P4Mdn** | **P5Mdn** | **P6Mdn** | **Mdn6-Mdn5** |
| --- | --- | --- | --- | --- | --- | --- | --- | --- | --- | --- |
| DMD | del 50-52 | distal | 87 | 553 | 547 | 492 | 545 | 531 | 554 | 23 |
| DMD | del 45 | distal | 87 | 541 | 625 | 598 | 637 | 605 | 652 | 47 |
| DMD | exon duplic 62 | distal | 100 | 613 | 535 | 527 | 685 | 678 | 668 | -10 |
| DMD | del 48-50 | distal | 119 | 602 | 688 | 602 | 710 | 656 | 688 | 32 |
| DMD | exon duplic 55 | distal | 119 | 477 | 467 | 494 | 582 | 508 | 527 | 19 |
| DMD | del 46-53 | distal | 89 | 594 | 594 | 594 | 617 | 641 | 578 | -63 |
| DMD | del 45-52 | distal | 100 | 570 | 516 | 500 | 516 | 594 | 531 | -63 |
| DMD | del 45-52 | distal | 95 | 594 | 531 | 469 | 516 | 539 | 500 | -39 |
| DMD | del 45 | distal | 93 | 516 | 719 | 492 | 581 | 505 | 526 | 21 |
| DMD | del 46-48 | distal | 108 | 461 | 438 | 453 | 522 | 375 | 414 | 39 |
| DMD | del 46-48 | distal | 108 | 562 | 625 | 562 | 536 | 614 | 609 | -5 |
| DMD | del 46-50 | distal | 87 | 688 | 578 | 625 | 531 | 625 | 641 | 16 |
| DMD | del 43 | proximal | 85 | 453 | 484 | 547 | 484 | 531 | 562 | 31 |
| DMD | del 45-50 | distal | 95 | 484 | 609 | 625 | 695 | 766 | 625 | -141 |
| DMD | del 45 | distal | 89 | 750 | 715 | 670 | 809 | 787 | 765 | -22 |
| DMD | del 52 | distal | 106 | 686 | 672 | 546 | 640 | 623 | 594 | -29 |
| DMD | del 49-50 | distal | 122 | 586 | 617 | 688 | 668 | 703 | 686 | -17 |
| DMD | del 44 | proximal | 98 | 728 | 699 | 687 | 762 | 689 | 714 | 25 |
| DMD | del 3-7 | proximal | 106 | 691 | 668 | 617 | 738 | 594 | 660 | 66 |
| DMD | del 44 | proximal | 108 | 498 | 482 | 500 | 504 | 527 | 504 | -23 |
| DMD | del 20-44 | distal | 100 | 523 | 660 | 656 | 648 | 676 | 582 | -94 |
| DMD | del 22-29 | proximal | 111 | 637 | 693 | 775 | 709 | 746 | 900 | 154 |
| DMD | del 8-41 | proximal | 101 | 531 | 484 | 703 | 688 | 576 | 578 | 2 |
| DMD | del 3-4 | proximal | 89 | 678 | 698 | 724 | 742 | 662 | 646 | -16 |
| DMD | del 6100 del Cn exon 42 | proximal | 93 | 515 | 559 | 551 | 545 | 576 | 578 | 2 |
| DMD | punt del G1616A 9 | proximal | 111 | 594 | 531 | 469 | 516 | 539 | 500 | -39 |
| DMD | del 45 | distal | 100 | 547 | 538 | 779 | 738 | 668 | 609 | -59 |
| DMD | exon duplic 15-22 | proximal | 108 | 461 | 476 | 474 | 531 | 554 | 516 | -38 |
| DMD | del c.2827del c ex 22 | proximal | 128 | 531 | 656 | 605 | 578 | 469 | 641 | 172 |
| DMD | exon duplic 8-47 | proximal | 130 | 625 | 623 | 617 | 625 | 672 | 641 | -31 |
| DMD | del 10-11 | proximal | 128 | 578 | 672 | 703 | 680 | 670 | 621 | -49 |
| TD |  |  | 103 | 641 | 609 | 545 | 527 | 459 | 476 | 17 |
| TD |  |  | 108 | 506 | 543 | 465 | 438 | 521 | 592 | 71 |
| TD |  |  | 100 | 609 | 553 | 594 | 672 | 562 | 678 | 116 |
| TD |  |  | 103 | 445 | 594 | 516 | 539 | 453 | 605 | 152 |
| TD |  |  | 108 | 625 | 693 | 732 | 732 | 734 | 750 | 16 |
| TD |  |  | 110 | 516 | 516 | 562 | 508 | 531 | 547 | 16 |
| TD |  |  | 129 | 438 | 482 | 492 | 443 | 461 | 467 | 6 |
| TD |  |  | 110 | 516 | 529 | 545 | 469 | 459 | 480 | 21 |
| TD |  |  | 99 | 625 | 625 | 609 | 551 | 562 | 688 | 126 |
| TD |  |  | 100 | 672 | 609 | 625 | 629 | 672 | 762 | 90 |
| TD |  |  | 90 | 547 | 537 | 576 | 562 | 531 | 570 | 39 |
| TD |  |  | 110 | 530 | 545 | 538 | 519 | 500 | 578 | 78 |
| TD |  |  | 90 | 514 | 576 | 625 | 562 | 551 | 553 | 2 |
| TD |  |  | 100 | 631 | 641 | 576 | 637 | 633 | 719 | 86 |
| TD |  |  | 109 | 586 | 529 | 599 | 531 | 515 | 547 | 32 |
| TD |  |  | 111 | 748 | 719 | 757 | 701 | 748 | 789 | 41 |
| TD |  |  | 92 | 709 | 631 | 599 | 570 | 609 | 615 | 6 |
| TD |  |  | 108 | 609 | 594 | 656 | 584 | 662 | 719 | 57 |
| TD |  |  | 91 | 719 | 652 | 652 | 695 | 654 | 668 | 14 |
| TD |  |  | 109 | 578 | 562 | 554 | 570 | 640 | 584 | -56 |
| TD |  |  | 90 | 594 | 605 | 617 | 631 | 625 | 594 | -31 |
| TD |  |  | 90 | 609 | 594 | 652 | 609 | 547 | 648 | 101 |
| TD |  |  | 127 | 609 | 648 | 625 | 609 | 562 | 656 | 94 |
| TD |  |  | 110 | 648 | 568 | 576 | 605 | 537 | 621 | 84 |
| TD |  |  | 110 | 406 | 438 | 453 | 476 | 474 | 500 | 26 |
| TD |  |  | 100 | 529 | 531 | 574 | 508 | 506 | 516 | 10 |
| TD |  |  | 100 | 516 | 538 | 623 | 577 | 530 | 578 | 48 |
| TD |  |  | 120 | 438 | 461 | 547 | 459 | 469 | 514 | 45 |
| TD |  |  | 95 | 562 | 492 | 537 | 535 | 422 | 531 | 109 |
| TD |  |  | 125 | 625 | 574 | 560 | 547 | 554 | 578 | 24 |
| TD |  |  | 90 | 578 | 547 | 562 | 656 | 633 | 656 | 23 |
| TD |  |  | 90 | 584 | 574 | 560 | 547 | 559 | 568 | 9 |
| TD |  |  | 110 | 574 | 508 | 578 | 484 | 516 | 551 | 35 |
| TD |  |  | 107 | 724 | 662 | 584 | 609 | 594 | 607 | 13 |
| TD |  |  | 90 | 506 | 578 | 639 | 609 | 590 | 594 | 4 |

**Legend**

DMD, Duchenne Muscular Dystrophy; TD, Typical Development; del, deletion; punt del, puntiform deletion; exon duplic, exon duplication;
